# Tailored outpatient physiotherapy rehabilitation versus standardised usual care physiotherapy after revision total hip replacement: protocol for a randomised controlled feasibility trial

**DOI:** 10.1136/bmjopen-2026-120891

**Published:** 2026-06-24

**Authors:** Erin Hannink, Beth Fordham, Stephen Gerry, Antony J Palmer, Francine Toye, Elizabeth A Stokes, Alana Morris, Karen L Barker

**Affiliations:** 1 Physiotherapy Research Unit, Oxford University Hospitals NHS Foundation Trust, Oxford, UK; 2 Nuffield Department of Orthopaedics, Rheumatology, and Musculoskeletal Sciences, University of Oxford, Oxford, England, UK; 3 Centre for Statistics in Medicine, University of Oxford, Oxford, UK

**Keywords:** Gait, Hip, Randomized Controlled Trial, Feasibility Studies, Quality of Life

## Abstract

**Background:**

Total hip arthroplasty (THA) is a highly effective procedure for improving pain and function in patients with advanced joint degeneration; however, revision surgery may be required because of complications or implant wear. Revision THA (rTHA) has a higher in-hospital mortality rate, longer hospital length of stay, are at higher risk of re-revision surgery and have worse physical and mental health outcome measures compared with primary THAs. The introduction of specialist revision hubs also means that patients frequently travel further for surgery. No specific guidelines have been established for rehabilitation after rTHA, leading to large variations in practice and potentially inadequate provision. We hypothesise that delivering a tailored physiotherapy intervention will improve and sustain greater functional outcomes and health-related quality of life compared with standard protocols currently in place. The aim of this study is to determine the feasibility and explore the acceptability of a trial investigating the effectiveness of a tailored physiotherapy intervention after rTHA.

**Methods and analysis:**

Multicentre, parallel two-arm feasibility randomised controlled trial with an embedded qualitative study. A total of 60 participants will be recruited from at least four UK NHS secondary care hospitals and randomly allocated (1:1 ratio) to either the tailored physiotherapy rehabilitation (THRIVE) programme or a standardised usual care arm. Eligible adults will be undergoing a single or final stage rTHA and participating in outpatient physiotherapy. Feasibility outcomes include recruitment rate, retention rate, adherence rate, intervention fidelity, outcome measure completion and acceptability of the intervention. Research assessments consisting of patient-reported and performance-based measures will occur preoperatively (baseline), with follow-ups at 4 and 8 months postoperatively. Feasibility data will be analysed using descriptive statistics. The embedded qualitative study will include trial participants and physiotherapists from the THRIVE arm to explore their experience of the trial and understand measures to improve the delivery of a future trial.

**Ethics and dissemination:**

The study has received ethical approval (West of Scotland REC 25/WS/0080), and all participants will provide informed consent. It will assess trial feasibility while exploring operational and safety challenges, including recruitment barriers and the potential value of a hub-and-spoke model for delivering physiotherapy. Findings will be disseminated through trial registry reporting, peer-reviewed open-access publication, conference presentations and participant summaries, with reporting aligned to CONSORT guidelines for pilot and feasibility trials.

**Trial registration number:**

ISRCTN10649335.

**STRENGTHS AND LIMITATIONS OF THE STUDY**

- A multi-centre feasibility randomised controlled design with embedded qualitative study enables a robust assessment of trial processes and intervention acceptability.
- A theory-informed, tailored physiotherapy intervention incorporating behaviour change techniques enhances relevance and potential adherence in a complex patient group.
- The inclusion of comprehensive outcome measures supports planning of a future definitive trial.
- A feasibility study with a small sample size is not powered to evaluate clinical effectiveness.

## Introduction

 Total hip arthroplasties (THAs) are highly successful and are associated with considerable improvements in health-related quality of life, joint function and patient satisfaction^1^; however, THAs have a finite lifespan, and some fail early because of complications, such as infection, fracture or component loosening, requiring revision surgery. In the context of an ageing population and growing annual volumes of primary THA, revision THA (rTHA) rates are continuously increasing, corresponding to more than 9000 cases annually in the UK between 2023 and 2025.^1^,^6^ In addition, rTHAs impose a considerable resource burden on the health system.^7 8^

Patients who have undergone rTHA have a higher in-hospital mortality rate, longer hospital length of stay, are at higher risk of re-revision and have worse physical and mental health outcome measures compared with those who had undergone primary THA.^9^,^11^ Recovery trajectories after rTHA plateau at 3 months after surgery in self-reported functional outcomes (pain and function) and gait speed; however, the same measures continue to improve after primary THA until 12 months.^10^ The introduction of specialist revision hubs also means that patients frequently travel further for surgery.^10^

Clinical practice in primary THA rehabilitation has evolved in the past decade, with evidence supporting the discontinuation of hip precautions after surgery^12^; however, this is not the case for rTHA in which variable precautions, contraindications and weight bearing status are often directed by individual surgeons based on the complex nature of revision surgery. Physiotherapy provision after rTHA varies widely across the UK.^13^ Sometimes, patients are treated identically to a primary THR, receiving self-directed home exercise or no rehabilitation, leading to large variations in practice and potentially inadequate provision.^13 14^

The National Institute for Health and Care Excellence (NICE) guidelines for joint replacements, based on evidence from primary THA rehabilitation, recommend self-directed rehabilitation at home or supervised outpatient rehabilitation depending on operation type and specific needs.^15^ This resonates with more recent systematic reviews also supporting self-directed exercise after primary THA.^16 17^ However, reviews highlight a lack of research on supervised physiotherapy for subgroups of patients with lower baseline functional status, which is a clinical characteristic of patients undergoing rTHA.

The limited primary evidence for rehabilitation after rTHA emphasises the importance of comprehensive physiotherapy rehabilitation that can be adapted.^18 19^ Qualitative research suggests that patients waiting for revision surgery have preconceived expectations around their surgery and recovery, and postoperatively, they do not always feel supported.^14 20^ In part owing to their experience, biopsychosocial involvement of rehabilitation and the complexity of surgery, patients may potentially benefit from a tailored approach to maximise engagement and adherence.

We hypothesise that delivering a tailored physiotherapy intervention will improve and sustain greater functional outcomes and health-related quality of life compared with standard protocols currently in place.

### Objectives

The primary objective of this feasibility study is to estimate recruitment and retention within the study. The secondary objectives are to (1) appraise adherence to the intervention, (2) evaluate intervention fidelity, (3) evaluate outcome measures for a full trial, (4) confirm a definitive primary outcome for a full trial and (5) explore the experience of participants in the trial and clinicians delivering the intervention (table 1).

**Table 1:** Study objectives and outcome measures

	Objectives	Outcome measures
Primary	To analyse (a) recruitment to the study and (b) retention in the study.	Screening logs, eligible patients consented (%) and randomised (%). Logs of data collection (retention rate)
Secondary	To appraise adherence to the intervention.	Physiotherapy session attendance, retention rate and qualitative interviews.
	To evaluate intervention fidelity.	Delivery of all three main components of the intervention (exercise progression, gait re-training and education) as monitored on treatment logs and qualitative interviews.
	To evaluate outcome measures for a full trial.	Assessment of the completion rate of the patient-reported outcome measures and performance-based outcome measures and qualitative interviews with patients and physiotherapists.
	To confirm a definitive primary outcome for a full trial.	Completion rate of expected primary outcome for future full trial: Oxford hip score
	To explore the experience of participants in the trial and physiotherapists delivering the intervention.	Qualitative interviews

## Methods and analysis

### Trial design

**Figure 1:**
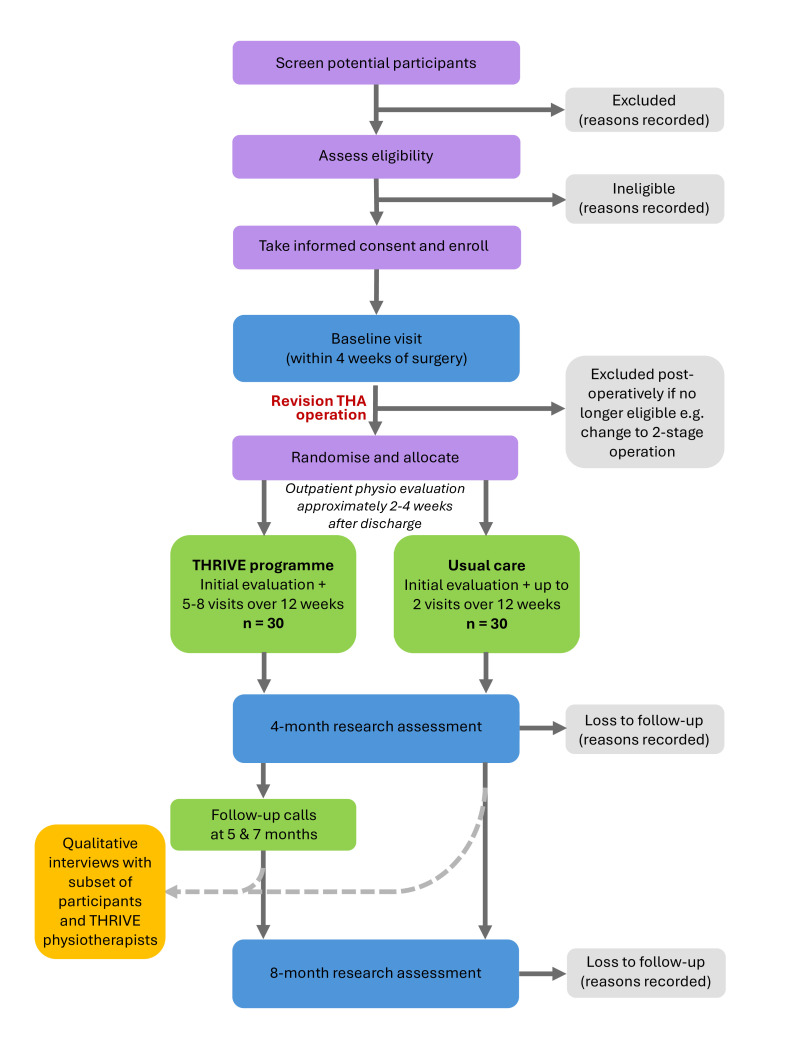
THRIVE trial flow chart. THA, total hip arthroplasty; THRIVE, tailored physiotherapy rehabilitation.

This study is a multi-centre, parallel two-arm feasibility randomised controlled trial (RCT) with an embedded qualitative study to explore acceptability. See figure 1 for an overview of the study design.

### Trial setting

The study will be conducted at a minimum of four NHS sites in England. The sites will be secondary care NHS hospitals that perform revision procedures. Participating centres include Oxford University Hospitals NHS Foundation Trust, North Bristol NHS Trust, St George’s University Hospitals NHS Foundation Trust, Royal National Orthopaedic Hospital NHS Trust and Cambridge University Hospitals NHS Foundation Trust. Sites may opt to establish a hub-and-spoke arrangement with their local community provider to deliver the physiotherapy intervention.

### Participant identification, recruitment and allocation

#### Eligibility

Site staff will screen adults on a preoperative list for a single-stage or final-stage rTHA.

The inclusion criteria are as follows:

- Participant is willing and able to give informed consent for participation in the study.
- Male or female, aged ≥18 years.
- Undergoing a single-stage rTHA or the final stage of a multi-stage rTHA.
- Independently mobile (with or without an assistive device).

The exclusion criteria are as follows:

- Planned lower limb surgery within 8 months.
- Conditions or comorbidities that make participation in an exercise programme unsafe (eg, severe acute or unstable cardiovascular or pulmonary disease, or undergoing radiotherapy or chemotherapy for cancer treatment).
- Other neurological or medical condition (eg, Parkinson’s disease, multiple sclerosis, cerebral palsy and conditions causing ataxia) that would prevent physical measures being collected.

### Recruitment

We will identify patients from the preoperative lists, and a preoperative clinician (eg, surgeon, nurse, physiotherapist or occupational therapist) will give potential participants a patient information sheet (PIS) during their face-to-face preoperative clinic appointment or send a PIS. After reading the PIS, if the patient expresses interest in participation and is willing to proceed, a site research team member will phone to confirm eligibility and arrange an appointment for the preoperative assessment visit. During this appointment, the site research clinician will take informed consent (online supplementary files: Example of Participant Consent form) and complete the baseline research. We will record the reasons for any exclusion at each step to meet our feasibility objectives.

### 3fRandomisation and allocation concealment

After the participants have undergone rTHA, the research clinician will re-assess their eligibility to participate. Participants may become ineligible if they require a further revision surgery or if their status and/or discharge destination has changed and impacts participation in outpatient physiotherapy. Eligible patients will be randomised by an unblinded research team member in a 1:1 ratio between the intervention and usual care groups using a web-based centralised randomisation database (SealedEnvelope), which will be managed by the trial manager. Stratified permuted block randomisation will be used, with stratification by sex and site. Blocks will be of random sizes. The statistician will oversee the allocation sequence produced by SealedEnvelope; no other members of the team will have access to it. By the nature of the intervention, patients and clinicians cannot be blinded to treatment once allocation has occurred. The research clinician collecting data from follow-up research visits will be blinded to treatment allocation. Additionally, data analysis will be conducted in a blinded fashion. Participants from both arms will complete the standard postoperative care pathway before being allocated to the treatment arm they were randomised to and scheduled for an outpatient physiotherapy appointment approximately 2–4 weeks post-operatively.

### Study arms

#### Comparator arm/usual care

Participants will receive a standardised usual care programme. Having completed consultations with stakeholders within centres that perform revision operations, usual care has been standardised to comprise an initial evaluation by a physiotherapist with a home exercise programme (approximately 60 min) and up to two follow-up sessions (30 min each) within 12 weeks, delivered in-person or remotely (depending on participant preference). This falls within the recommendation of the NICE guidelines for THA rehabilitation.

#### Intervention arm/tailored physiotherapy rehabilitation programme

**Figure 2:**
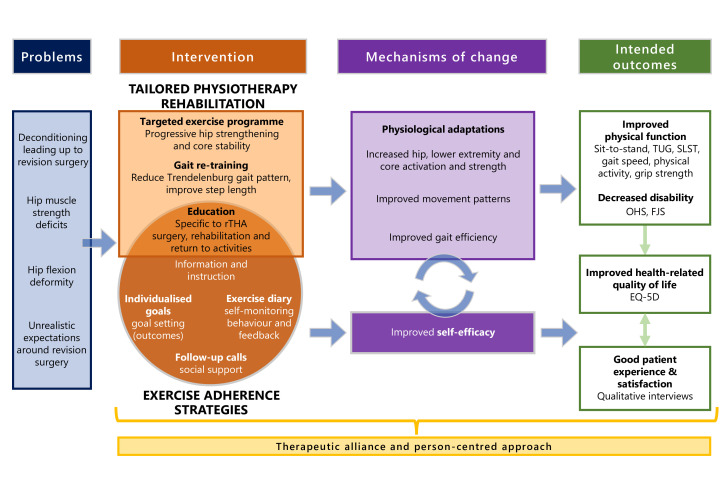
THRIVE Theory of change model. EQ-5D, EuroQol-5D; FJS, Forgotten Joint Score; OHS, Oxford Hip Score; rTHA, revision total hip arthroplasty; SLST, single leg stance; TUG, timed-up and go; THRIVE, tailored physiotherapy rehabilitation.

Our tailored physiotherapy rehabilitation (THRIVE) programme will involve targeted progressive strengthening, gait re-training and education. The delivery of the physiotherapy elements will be underpinned by the Capability, Opportunity, Motivation-Behaviour (COM-B) model and include evidence-based behaviour change techniques to encourage adherence.^21^ Owing to the complexity of the patient group, who will present with multiple co-morbidities and environmental factors to account for, the intervention will be delivered flexibly while maintaining the primary tenets of the intervention.^22^ In our theory of change model, we outline the interactions between problems in this patient group, our tailored physiotherapy intervention, the mechanism of change and the intended participant outcomes (figure 2). The THRIVE programme will comprise an in-person physiotherapy evaluation (approximately 60 min) and 5–8 follow-up sessions (30 min each) delivered in-person or remotely over a 12-week period. Additionally, there will be two follow-up phone calls (15 min each) 5 and 7 months postoperatively.

#### Targeted progressive strengthening

Targeted progressive strengthening will focus on hip muscle strengthening and core stability to improve functional activities.^23^ Due to preoperative de-conditioning compounded by the physical damage to tissues during surgery, postoperative strengthening is critical.^24 25^ Improving strength involves both neuromuscular adaptations (improving the signal from the brain to activate the muscle) and muscle hypertrophy (increasing the size of the muscle fibres). In addition to restoring strength for functional activities such as getting in and out of bed, up and down from a chair, walking to the shops, gardening and other physical recreation and hobbies, muscles around the hip joint provide active stabilisation that protects the prosthetic joint. Targeted progressive strengthening will be milestone-based progression, as opposed to strictly time-based progression which does not consider individual patient presentation and complexities associated with a revision surgery. The duration of the intervention is particularly important to allow for strengthening adaptations.

#### Gait re-training

Working in conjunction with progressive strengthening principles, gait re-training will aim to restore gait pattern, gait speed and confidence. After rTHA, fixed hip flexion and deficient hip abduction remain problematic, leading to gait instability.^26^ The physiotherapist will focus on re-training and stabilising gait as strength and range of motion improve. Progression will be tailored to the participant’s goals and environmental needs.

#### Education

Concurrently with participants’ physical rehabilitation, education delivered as part of the THRIVE programme will be evidence-based and individualised to address issues around rTHA, including any restrictions or precautions. Advice will be provided for returning to physical activity and participant-specific activities identified in their rehabilitation goals. Participants will be given an information booklet to guide and prompt questions to facilitate participant education.

#### Exercise adherence strategies

Importantly, integrated within the THRIVE programme will be exercise adherence strategies to support transition to self-management after the active 12-week intervention. An essential part of taking on an exercise programme during rehabilitation after major surgery is adhering to the programme.

We are asking our patients to make a significant health change by taking on an intensive exercise programme and performing exercises at home. Numerous factors impact on exercise adherence and engagement in people with long-term musculoskeletal health conditions such as hip osteoarthritis and joint arthroplasty. For example, people may feel uncertain about possible benefits or feel hesitant because they lack knowledge or skill; they may not feel confident in their physical ability because of comorbidities or may have negative views about treatment (fear of pain, dislocation or falling); there may also be financial barriers that impact engagement.

Guided by the COM-B model of behaviour change, several proposed mechanisms that might mediate or moderate patient’s adherence to physiotherapy were identified. We identified that an evidence-based mechanism of increasing self-efficacy (an individual’s belief in their capacity to complete a task or achieve a goal) will improve adherence to the exercise programme.^27^,^30^ We have integrated several exercise adherence strategies to enhance self-efficacy that are built on behaviour change techniques (BCTs) to target common barriers and facilitators to exercise specific to our clinical population.^20 31^ Our four approaches are as follows:

1. *Goal setting:* physiotherapists will discuss with participants to establish patient-led, values-based goals to guide and progress the physical intervention.
2. *Education:* in addition to education being a central part of the physiotherapy intervention, it serves a dual purpose as a BCT by providing information about health consequences and instruction on how to perform the exercise programme.
3. *Self-monitoring and feedback:* participants will use an exercise diary to monitor their exercise behaviour at home; these diaries will also guide the physiotherapist’s feedback and progression during follow-up sessions.
4. Social support: physiotherapists will complete two 15 min follow-up phone calls after the 12-week intervention (5 and 7 months) to support and motivate continued exercise adherence behaviour.

The incorporated approaches have been used in similar clinical populations and in physiotherapy intervention research, therefore demonstrating their practicality and acceptability among clinicians and patients. The use of multiple approaches at different points during the active intervention and follow-up periods will contribute to sustained adherence to the prescribed exercise programme, consequently improving and sustaining functional outcomes. In addition, these approaches will help tailor the physiotherapy programme and contribute to the holistic approach and *therapeutic alliance,* which can improve adherence.^32 33^ Within musculoskeletal physiotherapy, a strong therapeutic alliance or ‘working relationship between a patient and a therapist’, can facilitate exercise and physical activity adherence and has the potential to grow and improve outcomes over the 12-week duration.

### Clinician training

Physiotherapists will be trained in the details of the protocol and how to deliver the respective treatment arms. Physiotherapists delivering the THRIVE programme will be different from the physiotherapists delivering usual care, and they will receive separate training. We will also instruct physiotherapists in each arm to refrain from discussing participants and treatments to prevent contamination. Training for physiotherapists delivering the THRIVE programme will include an overview of the trial, specific instructions for delivery of the THRIVE programme, information around mixing and blinding issues and a summary of documentation and forms. Training for physiotherapists delivering usual care will cover the same topics except for instructions specific to the THRIVE programme arm.

### Outcomes

#### Feasibility outcomes

The feasibility outcomes for this study are listed in table 1. Study objectives: data for these outcomes will be recorded on screening and participant logs tracking participation and reasons for decline or loss to follow-up at each stage from screening to the end of study. Qualitative interviews will explore acceptability of the trial from the participant and physiotherapist perspective. Participant interviews will take place after the intervention is complete, and interviews with physiotherapists delivering the THRIVE programme will take place after the final follow-up call has taken place.

#### Clinical outcomes

Our clinical outcomes include six self-reported questionnaires, five clinician-completed physical assessments and a resource use diary (table 2. Clinical outcome measures). The self-reported measures include the Oxford hip score,^34^ Forgotten Joint Score,^35^ EuroQol 5-Dimension 5-Level (EQ-5D-5L) measure of health-related quality of life,^36^ University of California, Los Angeles activity scale^37^ and Self-efficacy for Exercise.^38^ The physical outcome measures include the 4-meter walk test,^39^ 30-s chair stand test,^40^ timed up and go test,^41^ single-leg stance test^42^ and hand grip strength.^43^

**Table 2:** Clinical outcome measures

Outcome measure	Outcome description	Measurement scale
Oxford hip score	12-item patient-reported measure for hip pain and function in patients undergoing total hip replacement.	Total score from 0 (worst possible hip function) to 48 (best possible hip function)
Forgotten Joint Score	12-item patient-reported measure for joint awareness after surgery.	Total score from 0 (constant awareness/worst outcome) to 100 (never aware/best outcome)
EQ-5D-5L	5-dimension health-related quality of life measure and visual analogue scale (VAS) of current general health rating.	Value set applied to the responses to each level of each dimension, from 0 (death) to 1 (full health) and <0 represent health states worse than death. VAS is a vertical scale from 0 (worst health imaginable) to 100 (best health imaginable)
UCLA Activity Scale	Single-item 10-point scale measuring physical activity levels.	Total score from 1 (lowest level of activity) to 10 (highest level of activity)
Self-efficacy for exercise	Self-efficacy barriers to exercise	Total score from 0 (not confident at all) to 90 (very confident)
4- meter walk test	Normal gait speed	Measured in seconds and converted to seconds/metre; measured twice
30-s chair stand test	Functional lower extremity strength	Measured in number of complete stands
Timed up and go test	Balance and walking ability	Measured in seconds to complete task; measured twice
Single-leg stance test	Balance tested on both sides	Measured in seconds; two tests on each side
Hand grip strength test	Grip strength	Measured in kg by handheld dynamometer; three tests on each side
Resource use diary	Healthcare resource use and family social support	Quantity of healthcare and social/family resources consumed

EQ-5D-5L, EuroQol 5-Dimension 5-Level; UCLA, University of California, Los Angeles.

### Data collection

Feasibility data will be collected during screening, recruitment, eligibility re-assessment and randomisation, during the 12- week intervention period, at 4 months postoperative, at 5- and 7-months postoperative for the THRIVE programme arm, and at 8 months postoperative. Outcome measures will be collected at three timepoints: baseline (preoperative), 4 months postoperative and 8 months postoperative (table 3). Schedule of study procedures. Follow-up questionnaires at 4 and 8 months will be posted to participants who do not attend the in-clinic follow-up research visit. Information from Protocol Deviation forms will be collected ad hoc and patterns and frequencies of deviations will be investigated. Qualitative interviews will be conducted with participants following completion of their intervention. Qualitative interviews with treating clinicians from the THRIVE programme arm will be conducted after they have completed all treatments for all participants.

**Table 3:** Schedule of study procedures

Procedures	Pre-enrolment	Preoperative baseline	Postoperative		4 months follow-up	8 months follow-up	Post-intervention
	Screening	Visit 1	After operation	Intervention period	Visit 2	Visit 3	Qualitative interviews
Potential participant identification and screening	x						
Provision of PIS	x						
Eligibility assessment	x		x				
Informed consent		x					
Outcome questionnaire completion		x			x	x	
Physical assessment by research staff		x			x	x	
Randomisation			x				
Letter sent to GP informing study involvement			x				
Referral to physiotherapy (based on allocated arm)			x				
Trial interventions delivered				x			
Treatment log completed by physiotherapist (THRIVE intervention)				x			
Treatment log completed by physiotherapist (usual care)				x			
Home exercise diary (THRIVE intervention)				x			
Healthcare resource use and family social support diary				x	x	x	
Follow-up phone calls for THRIVE intervention				x			
Adverse events recorded				x	x	x	
Qualitative interviews with selected sample							x

GP, general practitioner; PIS, patient information sheet; THRIVE, tailored physiotherapy rehabilitation.

### Data management

Data will be collected using a mix of electronic and paper forms. Data will be pseudonymised using patient identification and stored on NHS servers in encrypted files. Data will be entered centrally into a REDCap database where data provenance will be documented. Qualitative interviews will be transcribed, de-identified and pseudonymised. Participant contact details will be deleted when no longer required as part of the study (following posting of the summary of study results). This will be within 12 months of the end of the study. Additional information regarding data management can be found within the Data Management and Access Plan.

### Statistics and data analysis

#### Sample size calculation

As this is a feasibility study that does not aim to assess treatment effects, we have not undertaken a formal power sample size calculation. A minimum of 60 participants (≥30 in each arm) will be recruited, as recommended to estimate key design parameters in a feasibility RCT and establish the study sample size for a definitive trial.^44^ The qualitative sub-study will include up to 24 participants from the main trial (up to 16 from the THRIVE intervention group and up to eight from the usual care group). Additionally, we will interview up to eight clinicians who delivered the THRIVE intervention. These qualitative samples are aligned with interpretive qualitative research methods that do not rely on statistical representation. Our sampling strategy will be influenced by the principles of Information Power.^45^

#### Statistical analysis plan

Primary analysis will evaluate the feasibility of conducting the study. The feasibility outcomes (eg, recruitment rate, consent proportion, physiotherapy session attendance and retention rate) will be described by randomisation group using frequency, proportions and figures, where appropriate.

We will use progression criteria to assess the feasibility of a future definitive trial using a ‘traffic light’ system (table 4. Trial progression criteria). These quantitative progression criteria will be considered in combination with qualitative findings to guide decision making and trial design. Green (go) indicates feasible with the current procedures, amber (amend) indicates feasible if there is modification to one or more components of the protocol and red (stop) indicates a definitive trial would not be feasible without significant changes to protocol.

**Table 4:** Trial progression criteria

Criteria	Green/go	Amber/amend	Red/stop
Recruitment	**≥60** eligible participants recruited within 10 months	**45-59**	**<45**
Intervention fidelity	**≥75%** of participants receive the allocated intervention per protocol	**50%–74%**	**<50%**
Adherence	**≥75%** of THRIVE intervention participants have at least five follow-up sessions delivered	**50%–74%**	**<50%**
Outcome data completion rate	**≥80%** of participants have 8-month outcome data	**≥60%–79%**	**<60%**
Outcome measure use in full trial	**Two or more** outcomes suitable for full trial	**One outcome** suitable for full trial	**No outcomes** suitable for full trial
Intervention implementation	Delivery of intervention judged **strongly feasible** by qualitative data	**Feasible**	**Possibly feasible**

THRIVE, tailored physiotherapy rehabilitation.

The characteristics of the randomised groups will be described using mean and SD, or frequency and proportions, as appropriate.

The outcome assessments, including patient-reported outcome measures and performance-based measures, will be reported in a descriptive fashion, with no formal assessment of treatment effect between groups since this is a feasibility study. We will summarise outcomes overall and in each group. Completeness and variability of the outcome measures will be used together as a guide to help determine the best outcome for a future main study. We will visualise the change in outcomes over time within groups using plots. All analyses will follow intention-to-treat principles. We will report the amount of missing data for each outcome and summarise patterns of missingness.

### Health economics analysis

An economic evaluation would be an integral component of a future full trial and would assess the cost-effectiveness of tailored physiotherapy rehabilitation after rTHA compared with usual care, taking an 8-month (within trial) time horizon. The main outcome measure would be quality-adjusted life years, estimated using EQ-5D-5L.

In this feasibility study, the health economics component aims to test the practicality of collecting resource use and outcome data. We will take an NHS and personal social services perspective but will also collect data to assess whether there are substantial costs to patients and their families and whether a wider perspective should be considered in the full trial. Resource use will be recorded from the first physiotherapy appointment, with intervention-related data captured in trial case report forms and other health, social care and family support costs collected via participant resource use diaries at 4 and 8 months postoperatively. Participants will complete the EQ-5D-5L at baseline at 4 and 8 months postoperatively. For each time point, the proportion of participants followed up and data completeness for resource use and outcomes will be reported descriptively.

### Safety reporting

The principal investigators are responsible for assessing all adverse events (AEs) and categorising seriousness, expectedness and relatedness. AEs will be defined and reported according to Good Clinical Practice guidelines.

### Embedded qualitative study

The qualitative study will include trial participants and physiotherapists and aims to explore their experience within the trial and understand measures to improve the delivery of a future trial.

#### Qualitative study design

Semi-structured interviews will be conducted by experienced qualitative researchers following completion of participation in the study. Analysis will follow the steps of Braun and Clarke’s reflexive thematic analysis.^46^

#### Sampling and recruitment

Patient participants will be purposefully sampled to include participants from a range of trial sites, of both sexes, with different indications for surgery and from a range of socio-economic contexts to gather a range of perspectives. Our sampling strategy will be influenced by the principles of information power that consider the aim of the study, sample specificity, use of established theory, quality of dialogue and analysis strategy, which is compatible with reflexive thematic analysis. Participants will be recruited from the pool of trial participants who have consented to be contacted about the qualitative study. Potential participants for the qualitative studies will be provided a PIS for the qualitative study and given time to consider participation.

#### Interview data collection and analysis

All participants will be asked to give their informed consent for this qualitative study before their interview. Interviews will be conducted by an experienced qualitative researcher within 4 weeks of participants’ final contact with a physiotherapist as part of the trial. We will collect data via semi-structured interviews conducted in person or remotely (online supplementary files: interview guides). We will record the interviews, then transcribe and de-identify transcripts. We will analyse the data using reflective thematic analysis.^46^

### Study management

A core team comprising the trial manager and chief investigators will be responsible for overseeing day-to-day study management and will meet at least once a fortnight. A trial management group (TMG) composed of the study co-applicants and our patient collaborator will meet four times per year aligning with study milestones. A trial steering committee (TSC) will be led by an independent chair and consist of three external members including an expert clinician, trialist and statistician. The group will meet two to three times per year. As this is a feasibility study, a data monitoring committee was not deemed necessary. Any data queries regarding quality and completeness will be directed to the TSC.

### Patient and public involvement

We have a patient and public involvement and engagement (PPIE) group composed of patients with lived experience. During the development of the interventions and trial design, we consulted four patients in the group and one of their carers. Their feedback led to changes in our protocol, for example, we altered our physiotherapy follow-up delivery mode to ensure remote visits were an option. One member of the group serves as our patient collaborator in the TMG. The PPIE group will have ongoing input throughout the duration of the study, including study conduct and data interpretation, and importantly during dissemination.

### Trial status

Recruitment commenced on 3 September 2025. We have opened four sites. Current trial protocol (version 4.0) can be accessed at https://www.ouh.nhs.uk/media/hsofchhk/thrive-protocol.pdf.

### Trial governance

The trial is sponsored by Oxford University Hospitals NHS Foundation Trust. The sponsor has no role in the design of the study, data collection, analysis, interpretation or writing of the manuscript.

### Ethics and dissemination

A UK Research Ethics Committee has approved this study (West of Scotland REC 25/WS/0080). All participants will provide their informed consent to participate in the study. The study will investigate the feasibility of conducting this trial and will also examine closely the practical, operational and safety barriers that arise. This is a diverse clinical population, with high healthcare utilisation, and many revision operations are performed at regional hubs. Specialist referral pathways might be barriers for recruitment since outpatient physiotherapy is delivered at the surgical centre where we can ensure expertise and adequately experienced musculoskeletal physiotherapists. At one study site, we are trialling the hub-and-spoke model. Participants who live ‘out-of-area’ can receive their standardised usual care from their local community provider. This will allow us to understand if the model has the potential to increase recruitment, decrease clinical load on the secondary care centre, and if participants value receiving physiotherapy more locally. As we analyse our primary outcomes, which are recruitment and retention rates, we will focus on these potential issues and probe them in the qualitative interview study.

Trial results will be reported in the trial registry, published in an open-access academic journal, presented at relevant physiotherapy and orthopaedic conferences and shared in an accessible summary with the trial participants. The final report will detail amendments to the study protocol. The final manuscript will be reported according to the^47^ statement extension to randomised pilot and feasibility trials.^47^

## Supplementary material

10.1136/bmjopen-2026-120891 online supplemental file 1

10.1136/bmjopen-2026-120891 online supplemental file 2

## Data Availability

No data are available.
