# Supplementary material for: Tailored outpatient physiotherapy rehabilitation versus standardised usual care physiotherapy after revision total hip replacement: protocol for a randomised controlled feasibility trial
Source: BMJ Open. 2026 Jun 24;16(6):e120891. doi: 10.1136/bmjopen-2026-120891 (PMC13295798; doi:10.1136/bmjopen-2026-120891)
Supplement: online supplemental file 1 [file bmjopen-16-6-s001.pdf]

NHS
Oxford University Hospitals
NHS Foundation Trust

Local site Trust logo
(to be added)

Local Principal Investigator:
<name>
<contact details>
<contact details>
<contact details>

Site Code			*Screening number*			
		—	**S**			

PATIENT CONSENT FORM

Tailored pHysiotherapy RehabIlitation after reVision total hip rEplacement (THRIVE): a feasibility randomised controlled trial

	Please initial in box if you agree.	
1. I confirm that I have read the information sheet dated …………………… (version ……..) for this study. I have had the opportunity to consider the information, ask questions, and have these answered satisfactorily.		
2. I understand that my participation is voluntary and that I am free to leave the study at any time without giving any reason, without my medical care or legal rights being affected.		
3. I understand that relevant sections of my medical notes and data collected during the study may be looked at by individuals from Oxford University Hospitals NHS Foundation Trust, from regulatory authorities, <and from the local NHS Trust>, where it is relevant to my taking part in this research. I give permission for these individuals to have access to my records.		
4. I understand that if I lose capacity to consent during the study, I will be withdrawn from the study; identifiable data already collected with my consent would be kept and used in the study, and no further data would be collected or any other research procedures carried out.		
5. I agree to my General Practitioner being informed of my participation in the study.		
6. I agree to take part in this study.		
7. I agree to be contacted about the THRIVE trial participant interviews.	Yes	No

_____________________________ Name of participant
_____________________ Date
________________________________ Signature

_____________________________ Name of person taking consent
_____________________ Date
________________________________ Signature

When completed: 1 copy for participant; 1 for researcher site file (original);
1 copy to be kept in medical notes (if participant is a patient)
