# Supplementary material for: Tailored outpatient physiotherapy rehabilitation versus standardised usual care physiotherapy after revision total hip replacement: protocol for a randomised controlled feasibility trial
Source: BMJ Open. 2026 Jun 24;16(6):e120891. doi: 10.1136/bmjopen-2026-120891 (PMC13295798; doi:10.1136/bmjopen-2026-120891)
Supplement: online supplemental file 2 [file bmjopen-16-6-s002.pdf]

TOPIC GUIDE – PATIENT PARTICIPANTS

First, thanks so much for being part of the THRIVE study. We would like to hear your honest views about your experience so that we can improve and potentially do a large trial to see what works best for patients have revision hip surgery.

Guide for participants from the THRIVE intervention arm:

1. Please can you talk to me about why you had revision surgery?
2. Can you talk to me about your recovery and how things are now?
3. Can you talk to me about the impacts of revision surgery on your daily life, what you can do now, how you feel, and how it has impacted your family, social, work, hobbies, interests?
4. Can you tell me about your experience in the THRIVE study?
5. Can you talk to me about the tailored exercise programme (likes, dislikes, barriers)?
6. Can you talk to me about the educational elements (likes, dislikes, barriers)?
7. Can you talk to me about the gait training (likes, dislikes, barriers)?
8. Can you talk to me about the follow up sessions, including the phone calls (e.g. number/amount) (likes, dislikes, barriers)?
9. If you were designing the "best" physio after revision surgery, what would this look like?
10. If you were designing a trial to test the best treatment, what would you change (or keep)?
11. Can you think of any particular patients who would benefit from THRIVE (and who might not)?
12. Did anything unexpected or worrying happen or did you feel uncomfortable about anything during the study?
13. Would you make any changes to communication or information from your physiotherapist, or suggest how best to do this?
14. Did you have any remote physiotherapy and what do you think about it?
15. Can you talk to me about the assessments that you did (walking speed, walking and turning, sit to stand test, balance, grip strength)?
16. What did you think about the questions asked in the questionnaires?
17. What is the outcome that matters most to you following your surgery?

Guide for participants from the Usual Care arm:

1. Please can you talk to me about why you had revision surgery?
2. Can you talk to me about your recovery and how things are now?
3. Can you talk to me about the impacts of revision surgery on your daily life, what you can do now, how you feel, and how it has impacted your family, social, work, hobbies, interests?
4. Can you tell me about your experience in the THRIVE study?
5. Can you talk to me about the physiotherapy you received (likes, dislikes, barriers)?
6. If you were designing the "best" physio after revision surgery, what would this look like?
7. If you were designing a trial to test the best treatment, what would you change (or keep)?
8. Can you think of any particular patients who would benefit from being a part of the THRIVE study (and who might not)?
9. Did anything unexpected or worrying happen or did you feel uncomfortable about anything during the study?
10. Would you make any changes to communication or information from your physiotherapist, or suggest how best to do this?
11. Did you have any remote physiotherapy? What do you think about it?
12. Can you talk to me about the assessments that you did (walking speed, walking and turning, sit to stand test, balance, grip strength)?
13. What did you think about the questions asked in the questionnaires?
14. What is the outcome that matters most to you following your surgery?

TOPIC GUIDE – CLINICIANS

First, thanks so much for being part of the THRIVE study. We would like to hear your honest views about your experience so that we can improve and potentially do a large trial.

1. Please can you tell me about your experience in the THRIVE study?
2. Can you talk to me about the differences between the THRIVE intervention and what your normal protocol for revision hips would be?
3. Can you talk to me about any barriers to recruiting patients into the study? (prompts: examples of particular patients, e.g. age, ethnicity, education)
4. Can you think of any ways to improve inclusive recruitment?
5. Can you talk to me about any organisational barriers to the THRIVE study?
6. Can you talk to me about any barriers based on your own professional decision-making?
7. From your view and imagining the patient's point of view...
    a. Can you talk to me about the tailored exercise programme (likes, dislikes, barriers)?
    b. Can you talk to me about the educational elements (likes, dislikes, barriers)?
    c. Can you talk to me about the gait training (likes, dislikes, barriers)?
    d. Can you talk to me about the follow up sessions and phone calls (e.g. number/amount) (likes, dislikes, barriers)?
8. If you were designing the large trial, what would you change (or keep)?
9. If you were designing the "best" physio for revision hip, what would this look like?
10. Which parts of the THRIVE intervention (progressive exercise, educational elements, gait training, adherence strategies) do you think are most effective, and how?
11. What do you think patients thought about the THRIVE intervention (what did they like and not like)?
12. Were there any particular patients who benefitted (and who did not)?
13. Did anything unexpected or worrying happen or did you feel uncomfortable about anything?
14. Would you make any changes to communication or information from the research study team, or suggest how best to do this?
15. Can you talk to me about the benefits (or not) of remote consultations?
16. Can you describe a patient who did really well?
17. Can you describe a patient who did not do so well?
18. What do you think are the most important outcome for a trial following hip revision?
19. Would you be willing to take part in this as a future trial (if not, what would we need to change)?
